# Leveling the playing field: evaluating measurement equivalence in MMIs between genders

**DOI:** 10.3389/fmed.2025.1639532

**Published:** 2025-08-28

**Authors:** Jaclyn Michele Szkwara, Amy Jean Bannatyne, Mustafa Asil, Belinda Craig, Jessica Stokes-Parish, Jo Bishop

**Affiliations:** Medical Program, Faculty of Health Sciences and Medicine, Bond University, Gold Coast, QLD, Australia

**Keywords:** admission processes, selection processes, diversity and inclusion, gender, equity, medical education, medical school admission

## Abstract

**Introduction:**

The selection process for medical schools plays a vital role in identifying candidates with the attributes and capabilities needed for success in medicine. Multiple Mini-Interviews (MMI) are widely used to assess non-cognitive attributes like communication, empathy, and ethical judgment. Ensuring their fairness and validity across diverse applicant groups is essential for equitable selection.

**Aims:**

This study aimed to investigate: (1) is there evidence to support the factorial validity of MMI structure; (2) whether non-cognitive attributes assessed by MMIs are consistently interpreted across gender groups; and (3) whether gender-related disparities exist in MMI performance.

**Methods:**

Data were drawn from applicants to an Australian Medical School across three selection cycles (2022–2024). Confirmatory Factor Analysis (CFA) was used to assess the dimensionality of MMI performance, with multiple competing models tested to identify the best-fitting structure. The selected model was then assessed for measurement invariance across gender using Multi-Group CFA. Once scalar invariance was established, latent mean comparisons were conducted to examine gender-related differences in MMI performance.

**Results:**

CFA indicated a well-fitting structure for MMIs, with a higher-order model emerging as the most appropriate representation across cohorts. Measurement invariance testing confirmed scalar invariance across gender groups, indicating that MMI non-cognitive attributes were demonstrated equivalently by males and females. Significant latent mean differences were identified, with female applicants consistently outperforming male applicants across all 3 years.

**Discussion:**

The results provided empirical support for the factorial validity and measurement fairness of the MMI across gender groups. However, the consistent gender-based performance differences highlight the need for continued research into potential sources of group disparities and how they may impact selections equity. The results are relevant for medical educators and policymakers committed to evidence-based and equitable selection processes.

## 1 Introduction

The selection process for medical schools plays a vital role in identifying candidates with the attributes and capabilities needed for success in medicine. In recent years, medical school selection processes have evolved significantly, transitioning from traditional academic selection models to multifaceted selection processes that evaluate non-cognitive attributes such as communication, empathy, and ethical judgment, that are considered essential for health professionals (1–6). With significantly more applicants than available places, medical schools have sought more robust processes that not only identify academically strong candidates but also include non-cognitive assessments (7, 8).

Given this reality, selection processes must be both rigorous and evidence-based to ensure that candidates admitted to medical schools possess the necessary attributes to succeed in medical training and practice. While academic performance remains a strong predictor of success in the early years of medical education (6, 47), additional qualities such as empathy, ethical reasoning, and interpersonal effectiveness, are equally vital in developing competent and compassionate healthcare professionals (9). The incorporation of Multiple Mini-Interviews (MMI) (2) into selection processes offers a well-supported approach to evaluating these attributes. MMIs have demonstrated reliability and predictive validity in ranking candidates based on non-cognitive attributes essential for clinical excellence (2, 3, 6, 10, 11).

Originating in Canada, MMIs are now widely used internationally as a standardized and reliable method for assessing medical school applicants’ non-cognitive attributes (2, 4, 11, 12). MMIs are designed to evaluate candidates beyond their academic credentials, focusing on interpersonal and intrapersonal skills that are essential for success in medicine (13). The format shares conceptual similarities with the Objective Structured Clinical Examination (OSCE), which is frequently used to assess the knowledge and practical competencies of medical students. While OSCEs test applied clinical skills, MMIs are designed to evaluate how applicants respond to structured tasks that reveal their non-cognitive attributes (2, 4, 13).

MMIs typically consist of a series of structured interview stations, each presenting candidates with a distinct scenario or task designed to assess specific non-cognitive attributes. Candidates rotate between stations, responding to prompts that may involve ethical dilemmas, role-playing exercises, or situational problem-solving tasks. Each interaction is timed, typically lasting between 6 and 10 minutes, and responses are evaluated against predetermined criteria to ensure consistency and fairness in assessment (1, 2). Unlike traditional panel interviews, which rely on the judgment of a single interviewer or panel evaluating one scenario (1, 2, 4, 47), MMIs engage multiple independent assessors across different stations to ensure more standardized evaluation reducing the impact of individual bias and improving interrater reliability (2, 11).

Importantly, MMIs do not aim to measure personality traits. Rather, they assess candidates’ behaviors and approach as they navigate structured tasks within a defined framework of professional expectations. In doing so, it is also hoped that selection processes including MMIs will identify suitable applicants without systematically advantaging or disadvantaging applicants of certain demographics. Research examining potential bias in MMI processes has produced mixed findings. Some studies suggest that female candidates tend to be evaluated more favorably by assessors in many (5, 14), but not all MMI processes (15). This may reflect broader gender-based expectations and stereotypes that portray women as warmer, more empathetic, and possessing stronger verbal communication skills than men (16, 17). If such stereotypes have some basis in actual skill differences, it is possible that female applicants are objectively stronger in non-cognitive attributes assessed in MMIs. However, there is also evidence that both male and female assessors tend to evaluate women as more positive than men outside of the context of selection (18). These evaluative biases, potentially shaped by stereotypes, may result in more favorable assessments of female candidates even when objective performance is equivalent. As such, it is important to establish whether there are gender differences in performance and if so, whether they reflect true differences in candidate ability or are instead because of biases introduced in the tool used to evaluate applicants.

Various approaches have been used to assess performance on MMI stations, including use of a single score (global rating), or an aggregate score based on station specific rubrics, or a general rubric/tool applied to all stations (19). Utilizing a consistent and well-defined framework to evaluate candidate’s performance is one strategy to mitigate assessor bias and ensure equitable assessment for all candidates. Fairness and transparency in medical school selection processes involve designing tools that assess candidate attributes in a consistent, unbiased manner regardless of gender, socioeconomic status, or ethnicity (1, 4, 5, 20).

Previous studies have consistently reported higher MMI scores for female applicants (7, 14, 48), raising important questions about whether these differences reflect true variation in attributes assessed or potential scoring biases. Previous studies on MMI dimensionality have reported mixed findings, with some supporting unidimensional models and others endorsing multi-group factor analysis (21, 22) (Leduc et al., 2017). To clarify these inconsistencies, researchers have applied generalizability theory (23) and multi-group confirmatory factor analysis (MG-CFA) to disentangle sources of reliable variance, revealing the MMIs inherently multidimensional structure (21, 22) (Leduc et al., 2017). To address this, we applied MG-CFA to test whether the Behaviorally Anchored Rating Scales (BARS) used in our MMIs function equivalently across gender. Establishing measurement invariance is essential, as noninvariance suggests that a construct may differ in structure or meaning across groups, rendering group comparisons invalid (24).

This study aimed to investigate: (1) is there evidence to support the factorial validity of the MMI structure at our medical school; (2) whether core attributes assessed in MMIs are consistently interpreted across gender groups; and (3) whether gender-related disparities exist in MMI performance. By exploring these aims, researchers can assess whether MMIs function as equitable selection tools or whether underlying biases affect their effectiveness. Addressing these concerns is essential in ensuring medical school selection processes continue to be fair, inclusive, and reflective of the diverse populations they serve.

## 2 Methods

### 2.1 Study design and setting

This retrospective, cross-sectional study used data from applicants to an Australian Medical School across three selection cycles (2022–2024). This time range was selected due to the relative stability of our selection processes during these years.

### 2.2 MMI process and scoring method

**Figure 1:**
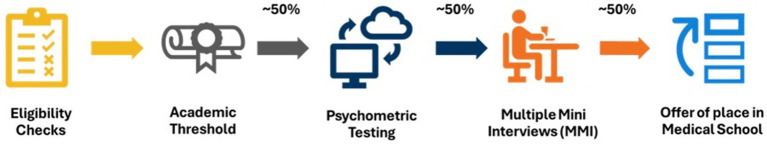
Overview of staged medical school selection process at our institution.

At our institution, applicants undertake MMIs as the final step in a staged selection process that includes four sequential and independent components: (1) eligibility checks (e.g., domestic student), (2) meeting a minimum academic threshold, (3) completion of proctored psychometric testing (ability-based emotional intelligence and self-report personality assessments), and (4) performance in MMI. Each stage is assessed independently, with no cumulative or weighted scoring across stages. Approximately 50% of applicants are excluded from the selection process at each stage. Importantly, it is MMI performance alone that determines whether an offer is made. See Figure 1 for an overview of the full selection process.

Consistent with previous literature, MMI stations at our institution are designed to evaluate non-cognitive attributes that are critical for future medical practice, including communication, ethical reasoning, empathy, and professionalism. MMIs comprise six stations (4 active and 2 rest), each lasting approximately 8 min, with a short transition period between stations (see Table 1 for further details). Each station presents candidates with a unique scenario or task aligned to specific non-cognitive attributes. These may involve ethical dilemmas, role-playing activities with a simulated participant, group activity tasks, or situational behavioral scenarios. Each MMI station is independently rated by two trained assessors (excluding the group station – see Table 1) using a Behaviorally Anchored Rating Scale (BARS) (25), which was developed specifically for our medical school through a three-round modified Delphi process (26, 27) of 13 experts.

**Table 1:** Overview of the MMI process at our institution across 2022–2024.

Year	# of active stations	# of rest stations	Broad station details	# of assessors	Timing	BARS Scoring
2022	4	2	Station 1 – role play with SP Station 2 – interview based on scenario video Station 3 – role play with SP Station 4 – interview based on scenario	2	1 min perusal 7 min response	7 key non-cognitive attributes (as described in text) 4-point Likert scale (1 = *not performed / unsatisfactory* to 4 = excellent)
2023	4	2	Station 1 – interview based on topic Station 2 – role play with SP Station 3 – interview based on scenario video Station 4 – group activity	2 (only 1 for S4: group activity)	1 min perusal 7 min response	7 key non-cognitive attributes (as described in text) 5-point Likert scale (1 = *not performed / unsatisfactory* to 5 = *above expectations*)
2024	4	2	Station 1 – interview based on topic Station 2 – role play with SP Station 3 – interview based on scenario video Station 4 – group activity	2 (only 1 for S4: group activity)	2 min perusal 6 min response	7 key non-cognitive attributes (as described in text) 5-point Likert scale (1 = *not performed / unsatisfactory* to 5 = *above expectations*)

BARS are a standardized scoring method to distinguish between a range of behaviors, as opposed to skills (25). In this type of scale, specific behaviors are identified that contribute to an overall rating of performance. BARS is typically developed by consensus methods to define the main dimensions of a particular role, clearly identifying the scale of performance (28). For example, Wright et al. (25) defined five dimensions that are a measure of teamwork, with explicit behaviors that demonstrate proficiency. In our case, we were looking to define the key behavioral attributes expected from prospective medical students at an Australian institution. Following a consensus seeking process, seven non-cognitive attributes were established with associated scales to measure non-cognitive attributes in prospective medical students.

The BARS provides assessors with clearly defined descriptors of performance for each score point, anchored in specific, observable behaviors across seven key non-cognitive attributes: (1) communication skills, (2) self-regulation, (3) effective team member, (4) adaptability, (5) analytical/critical thinking, (6) empathy, and (7) cultural capability. This allows interviewers to base ratings on observed candidate behavior rather than subjective impressions, thereby mitigating assessor bias (29). The use of the BARS across all MMI stations contributes to the fairness, transparency, and defensibility of the selection process (25, 28). It also ensures candidates are assessed against consistent criteria, regardless of which station or assessor they encounter.

Before participating in MMIs at our institution, assessors are provided with interviewer training resources and engage in interviewer training sessions to support standardization and reduce the potential for subjective bias. The aim is to familiarize interviewers with the structure of MMIs, how to deliver specific station scenarios, understand the BARS, and how to interpret the BARS for different scenarios. Interviewers are also provided with training on implicit biases that can influence evaluations of applicants (30).

In 2022, the BARS was scored on a 4-point Likert scale (1 = *not performed / unsatisfactory* to 4 = *excellent*). From 2023, a neutral anchor point was introduced, and the BARS was scored using a 5-point Likert scale (1 = *not performed / unsatisfactory* to 5 = *above expectations*). At each station, two interviewers independently evaluate candidates based on the seven non-cognitive attributes defined BARS framework. Scores for each station are aggregated into a total score. Where a score had been missed by an assessor or where there was only a single assessor, the average score for that station on that day the candidate attended was awarded and included in the aggregated total score.

MMIs at our institution do not employ a predefined threshold score to determine candidate success. Rather than adhering to a static benchmark, the annual cut score is established upon the performance distribution of candidates that had advanced to the next stage of the medical selection process. This data-driven approach allows the selection framework to accommodate year-to-year variability in candidate cohorts and maintain alignment with programmatic objectives and capacity constraints. While this method ensures contextual fairness, it also necessitates transparency in scoring procedures and rigorous post-hoc analysis to uphold reliability and equity in selection outcomes.

### 2.3 Study participants

Participants were applicants to our medical school who completed MMIs in 2022 (*N* = 404), 2023 (*N* = 428), and 2024 (*N* = 432). One applicant from each of the 2023 and 2024 cohorts identified their gender as “Other” and was excluded from gender-based analyses due to the small subgroup size. Gender distribution was relatively balanced across years, with a slight female majority. The proportion of undergraduate (high school graduates with no tertiary experience) applicants increased steadily, from 78% in 2022 to 85% in 2024. Demographic distributions by gender and undergraduate/postgraduate status are summarized in Table 2.

**Table 2:** Participant demographics by year and gender.

Year	Total N	Male (*n*, %)	Female (*n*, %)	UG (%)	PG (%)
2022	404	189 (47%)	215 (53%)	78%	22%
2023	428	206 (48%)	222 (52%)	84%	16%
2024	432	187 (43%)	245 (57%)	85%	15%

*N*, sample size; UG, undergraduate applicants (high school leaver or less than 1.5 years of tertiary study); PG, postgraduate applicants (completion of a previous tertiary degree or more than 1.5 years of tertiary study).

As an Australian private institution, students can access government supported loans for only a portion of the total fees. There is no financial aid available only First Nations applicants are eligible for fee-waiver scholarships. This means that applicants are generally from families representing the highest levels of socioeconomic advantage.

### 2.4 Statistical data analysis

Data were analyzed in three phases using SPSS and JASP 0.19.3 (31). The following steps were followed after conducting initial data checks.

#### 2.4.1 Dimensionality assessment

**Figure 2:**
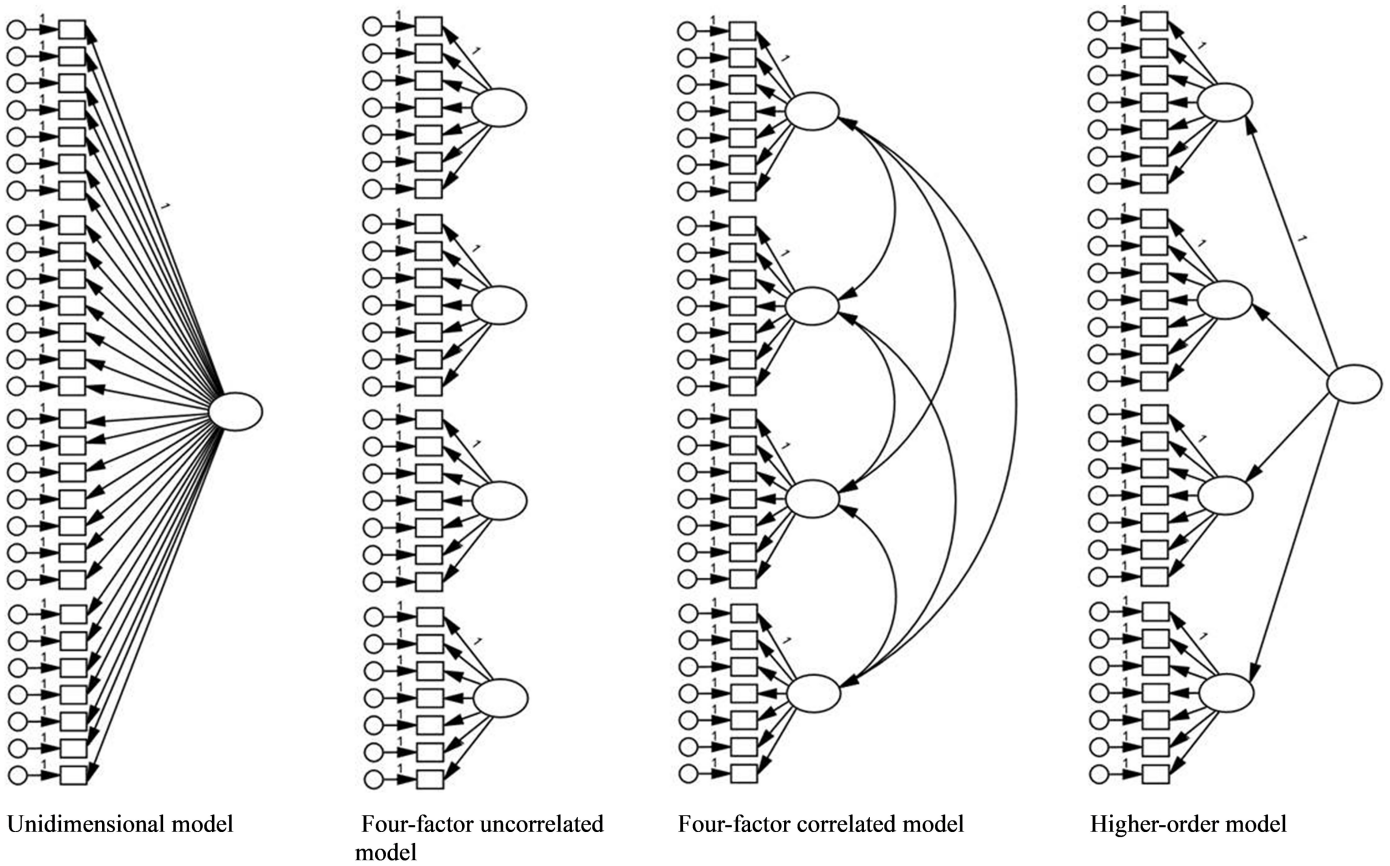
Confirmatory factor analysis – competing models.

Confirmatory Factor Analysis (CFA) was conducted separately for each year to examine the factor structure of MMI scores. Four competing models (Figure 2) were tested using the robust maximum likelihood (MLR) estimator:

1. Unidimensional model - assumes all MMI items reflect a single general construct.
2. Four-factor uncorrelated model - assumes each station taps into a distinct unrelated attribute.
3. Four-factor correlated model - assumes non-cognitive attributes measured at each station are distinct but interrelated.
4. Higher-order model - assumes station performances reflect specific attributes, underpinned by a broader construct (e.g., general ability).

Model fit was evaluated using standard indices in Structural Equation Modeling (SEM), including the root mean square error of approximation (RMSEA), comparative fit index (CFI), Tucker–Lewis index (TLI), and standardized root mean square residual (SRMR). Following conventional criteria, values of RMSEA and SRMR < 0.05, and CFI and TLI > 0.95 were considered indicative of good fit (32, 33). Due to sample size sensitivity, χ^2^ was reported but not used as a primary evaluation criterion.

To further assess construct validity and reliability, we examined standardized factor loadings, average variance extracted (AVE), coefficient alpha (*α*), and McDonald’s omega (*ω*). Given the limitations of α (e.g., assuming tau-equivalence), ω was prioritized as a more robust reliability indicator (34, 35).

#### 2.4.2 Measurement invariance (MI)

Multi-Group Confirmatory Factor Analysis (MG-CFA) was conducted to evaluate measurement invariance (whether the same underlying latent construct was measured equivalently) across applicant gender (Male vs. Female) within each year. A stepwise approach was applied:

1. Configural invariance - tests whether the same factor structure holds across groups, indicating that the constructs are conceptualized similarly across groups.
2. Metric invariance - tests whether factor loadings are equivalent, suggesting consistent interpretation of applicants’ demonstrated behaviors.
3. Scalar invariance - tests whether item intercepts are equivalent, allowing for valid comparisons of latent means across gender.

Model comparisons were based on changes in fit indices (ΔCFI and ΔRMSEA), following Chen (36). Changes of ≤ 0.010 in CFI and ≤ 0.015 in RMSEA were taken as evidence of invariance. Researchers generally agree that establishing scalar invariance is sufficient for supporting valid latent mean comparisons across groups (37–39). Without MI, any differences in scores could reflect measurement bias, not true differences in the underlying construct (40). In the context of medical school selection, testing measurement invariance is essential to ensure that assessment tools, such as MMIs, evaluate applicants consistently across demographic groups, thereby supporting fair and defensible selections decisions (38, 41, 42).

#### 2.4.3 Latent mean comparisons

Where scalar invariance was established, latent mean differences across gender were estimated using MG-CFA, with males as the reference group.

### 2.5 Ethical considerations

This research was approved by the Human Research Ethics Committee (AB03432) at the authors’ institution.

## 3 Results

### 3.1 Descriptive statistics

Assessor omissions for a non-cognitive attribute within the BARS framework occurred at a rate of 0.7% in 2022, 0.3% in 2023, and 0.0% in 2024. In cases where only a single assessor was present, 23.0% in 2022, and 0.0% in both 2023 and 2024, the station-specific average score, based on all candidates assessed on that day, was assigned for each of the seven non-cognitive attributes and incorporated into the aggregated total score. Table 3 presents descriptive statistics (means, standard deviations, ranges) for each MMI station by gender across all 3 years. Female applicants had consistently higher scores than males across all stations and years. Standard deviations were generally comparable between genders within each station and year, suggesting similar levels of score variability across groups.

**Table 3:** Descriptive statistics of MMI station scores by gender.

Year	Station	Gender	*M*	*SD*	Min–Max
2022	2022-S1	Male	18.57	4.41	9.00–28.00
	2022-S1	Female	20.45	4.00	9.50–28.00
	2022-S2	Male	22.21	3.99	10.50–28.00
	2022-S2	Female	23.33	3.50	14.00–28.00
	2022-S3	Male	19.66	4.69	7.00–28.00
	2022-S3	Female	21.36	4.07	7.00–28.00
	2022-S4	Male	20.54	5.03	7.00–28.00
	2022-S4	Female	22.87	4.04	9.00–28.00
2023	2023-S1	Male	26.62	5.88	10.00–35.00
	2023-S1	Female	28.86	4.58	14.50–35.00
	2023-S2	Male	25.12	5.74	8.00–35.00
	2023-S2	Female	27.05	4.86	9.50–35.00
	2023-S3	Male	25.71	4.82	14.00–35.00
	2023-S3	Female	27.30	5.00	9.50–35.00
	2023-S4	Male	27.83	4.64	8.00–35.00
	2023-S4	Female	28.89	4.18	13.00–35.00
2024	2024-S1	Male	27.55	4.61	12.00–35.00
	2024-S1	Female	28.82	4.14	12.00–35.00
	2024-S2	Male	23.78	6.22	9.00–34.50
	2024-S2	Female	25.75	5.55	10.50–35.00
	2024-S3	Male	26.99	4.91	10.00–34.50
	2024-S3	Female	28.46	4.15	14.00–35.00
	2024-S4	Male	27.78	5.97	7.00–35.00
	2024-S4	Female	28.93	4.22	12.00–35.00

MMI stations differ across years; that is, Station 1 in 2022 is not equivalent to Station 1 in 2023 or 2024. Station numbering is used solely for within-year identification.

### 3.2 Confirmatory factor analysis (CFA)

Model fit indices for all tested models are presented in Table 4. The unidimensional model showed poor fit across all cohorts. The four-factor uncorrelated model improved fit considerably but underperformed relative to the four-factor correlated and higher-order models. Both the correlated and higher-order models demonstrated excellent and nearly equivalent fit. Chi-square difference tests between the correlated and higher-order models indicated no significant loss of fit when adopting the more parsimonious higher-order structure (2022: Δχ^2^ = 1.733, Δdf = 2, *p* = 0.420; 2023: Δχ^2^ = 0.075, Δdf = 2, *p* = 0.963; 2024: Δχ^2^ = 2.475, Δdf = 2, *p* = 0.290). Given these results and the theoretical appeal of modeling a general ability factor underlying MMI station performance, the higher-order model was retained for subsequent invariance testing. In the four-factor correlated CFA models, factor correlations ranged from 0.13 to 0.42 in 2022, 0.16 to 0.36 in 2023, and 0.03 to 0.23 in 2024, indicating generally low to moderate inter-factor relationships across years.

**Table 4:** Goodness-of-fit indices for competing CFA models.

Year	Model	*χ^2^*	*df*	RMSEA	CFI	TLI	SRMR
2022	Unidimensional	7109.655	350	0.219	0.221	0.159	0.258
	4-Factor Uncorrelated	698.506	350	0.050	0.960	0.957	0.143
	4-Factor Correlated	587.381	344	0.042	0.972	0.969	0.040
	Higher-Order	589.272	346	0.042	0.972	0.969	0.042
2023	Unidimensional	5439.608	350	0.184	0.304	0.248	0.200
	4-Factor Uncorrelated	721.022	350	0.050	0.949	0.945	0.140
	4-Factor Correlated	607.669	344	0.042	0.964	0.960	0.038
	Higher-Order	607.990	346	0.042	0.964	0.961	0.038
2024	Unidimensional	6763.427	350	0.206	0.223	0.161	0.247
	4-Factor Uncorrelated	610.713	350	0.042	0.968	0.966	0.093
	4-Factor Correlated	560.444	344	0.038	0.974	0.971	0.037
	Higher-Order	563.035	346	0.038	0.974	0.971	0.040

Table 5 reports internal consistency estimates, AVE, and range of factor loadings. Across all years, internal consistency was high for each station (*α* and *ω* > 0.88), and AVE values exceeded 0.50, indicating good reliability and convergent validity. All standardized factor loadings were statistically significant (*p* < 0.05).

**Table 5:** Factor reliabilities, average variance extracted (AVE), and factor loadings.

Year	Factor	Coefficient α	Coefficient ω	AVE	Factor Loadings range
2022	2022-S1	0.938	0.940	0.697	First order: 0.714–0.897 Second order: 0.305–0.662
	2022-S2	0.925	0.925	0.640	
	2022-S3	0.945	0.946	0.715	
	2022-S4	0.943	0.944	0.706	
2023	2023-S1	0.936	0.937	0.682	First order: 0.622–0.859 Second order: 0.356–0.705
	2023-S2	0.932	0.933	0.670	
	2023-S3	0.926	0.928	0.650	
	2023-S4	0.887	0.891	0.542	
2024	2024-S1	0.928	0.928	0.650	First order: 0.735–0.890 Second order: 0.289–0.713
	2024-S2	0.950	0.951	0.734	
	2024-S3	0.925	0.926	0.643	
	2024-S4	0.927	0.928	0.648	

All first and second order factor loadings were statistically significant (*p* < 0.05). First-order loadings represent item-level indicators; second-order loadings represent the relationship between first-order factors and the general latent construct.

### 3.3 Measurement equivalence

As shown in Table 6, configural, metric, and scalar invariance were supported in all 3 years. ΔCFI and ΔRMSEA values fell within accepted thresholds, confirming that the MMI structure was interpreted equivalently across gender groups.

**Table 6:** Measurement invariance testing by gender.

Year	Model	*χ^2^*	*df*	CFI	ΔCFI	RMSEA	ΔRMSEA
2022	Configural	990.569	692	0.965	-	0.046	-
	Metric	1023.549	719	0.964	−0.001	0.046	0.000
	Scalar	1056.515	742	0.963	−0.001	0.046	0.000
2023	Configural	1003.507	692	0.957	-	0.046	-
	Metric	1053.541	719	0.954	−0.003	0.047	−0.001
	Scalar	1086.413	742	0.953	−0.001	0.047	0.000
2024	Configural	920.765	692	0.972	-	0.039	-
	Metric	935.761	719	0.973	0.001	0.037	−0.002
	Scalar	968.399	742	0.972	−0.001	0.038	0.001

### 3.4 Gender differences

Table 7 presents latent mean differences by gender. In all 3 years, females had significantly higher latent mean scores compared to males. While measurement invariance confirmed that the MMI measured non-cognitive attributes equivalently across gender, persistent latent mean differences indicate a systematic gender-related performance trend.

**Table 7:** Latent mean differences by gender and year.

Year	Unstandardised estimate (Female – Male)	Std. Error	*p*-value
2022	0.126	0.035	< 0.001
2023	0.171	0.038	< 0.001
2024	0.135	0.061	0.028

Latent means for the male group were fixed to zero in the models.

## 4 Discussion

This study examined the dimensionality, reliability, and measurement equivalence of MMI scores across three consecutive selection cycles, with a particular focus on gender-based fairness. By applying a robust psychometric framework, we provide empirical evidence supporting the structural validity and reliability of MMIs, while also identifying consistent gender-based performance trends that warrant further attention.

Across all 3 years, a higher-order factor structure best represented MMI station performance, with each station loading onto specific non-cognitive attributes that were, in turn, underpinned by a broader latent non-cognitive ability construct. The findings align with prior literature conceptualizing MMIs as multidimensional tools that evaluate distinct, but related non-cognitive attributes that contribute to performance as a medical student and future clinician (2). The high internal consistency values, strong factor loadings and AVE estimates across cohorts further support the reliability and convergent validity of the BARS used in our selection process. Notably, the four-factor correlated model used also demonstrated excellent fit, indicating that station-level scores retain value as discrete measures of specific non-cognitive attributes. Thus, depending on the intended purpose, institutions may reasonably use either composite scores or station-specific results.

Crucially, measurement invariance analyses confirmed that male and female applicants interpreted and responded to the MMI tasks similarly, and that the observed scores reflected equivalent measurement of non-cognitive attributes across gender. These findings provide strong evidence that the BARS functioned equivalently across gender and may reflect the contribution of the associated interviewer training protocols to supporting equitable assessment practices. Establishing measurement invariance is a necessary precondition for making valid group comparisons and is an often overlooked, yet vital component of evaluating fairness in selection tools (38, 39).

Notably, females scored significantly higher than males on latent MMI performance across all 3 years, suggesting a consistent gender-related performance pattern that warrants further exploration. This pattern mirrors trends in other MMI-based studies (4, 5, 14, 20, 43–46) and may reflect genuine gender differences in the non-cognitive attributes assessed such as communication, empathy, and teamwork. Importantly, since scalar invariance was established, these differences likely reflect true variation in performance rather than measurement bias.

Nonetheless, persistent gender differences raise important questions for medical school leadership when considering their selection policies and procedures. While MMIs are designed to assess attributes essential to successful progression through medical school and future practice, selection processes must also ensure that such tools do not inadvertently favor certain groups. Future research might explore whether these gender differences persist in longitudinal academic, clinical outcomes or future career choices, or whether they reflect modifiable differences in socialization, experience, or preparation for the MMI format.

### 4.1 Strengths, limitations and future research

This study has several strengths, including its multi-year design, robust analytic approach, and use of a structured, theoretically grounded scoring system. Moreover, the consistency of findings across the 3 years enhances the generalizability of the results. However, some limitations should be acknowledged. First, our data reflects the context of a single private institution that is predominately a school leaver/undergraduate entry medical school. As such, the findings may not extend to other MMI designs or applicant populations. Second, while the sample sizes were sufficient for CFA and MG-CFA, the exclusion of non-binary participants due to small subgroup size limited the inclusivity of the analysis. Future research should explore intersectional factors (e.g., gender, culture, age, socioeconomic background) and use mixed methods to better understand why gender differences in performance persist despite structural equivalence. Replication across different settings/institutions would also be beneficial.

## 5 Conclusion

The results provide strong psychometric support for the use of MMIs in medical school selection processes, confirming their structural integrity and fairness in terms of measurement across gender. While the MMI assessed non-cognitive attributes equivalently for male and female applicants, consistent gender-based differences in performance were observed. These findings underscore the importance of ongoing monitoring of performance patterns and further investigation into the factors contributing to these disparities. As medical schools continue to refine their selection processes, integrating psychometric validation and fairness analyses into routine practice can support more defensible and equitable decision making.

## Data Availability

The raw data supporting the conclusions of this article will be made available by the authors, upon reasonable request.
